# [^18^F]FDG uptake of bone marrow on PET/CT for predicting distant recurrence in breast cancer patients after surgical resection

**DOI:** 10.1186/s13550-020-00660-y

**Published:** 2020-06-30

**Authors:** Jeong Won Lee, Sung Yong Kim, Sun Wook Han, Jong Eun Lee, Hyun Ju Lee, Nam Hun Heo, Sang Mi Lee

**Affiliations:** 1 Department of Nuclear Medicine, International St. Mary’s Hospital, Catholic Kwandong University College of Medicine, Simgok-ro 100-gil 25, Seo-gu, Incheon, 22711 South Korea; 2 Department of Surgery, Soonchunhyang University Cheonan Hospital, 31 Suncheonhyang 6-gil, Dongnam-gu, Cheonan, Chungcheongnam-do 31151 South Korea; 3 Department of Pathology, Soonchunhyang University Cheonan Hospital, 31 Suncheonhyang 6-gil, Dongnam-gu, Cheonan, Chungcheongnam-do 31151 South Korea; 4 Clinical Trial Center, Soonchunhyang University Cheonan Hospital, 31 Suncheonhyang 6-gil, Dongnam-gu, Cheonan, Chungcheongnam-do 31151 South Korea; 5 Department of Nuclear Medicine, Soonchunhyang University Cheonan Hospital, 31 Suncheonhyang 6-gil, Dongnam-gu, Cheonan, Chungcheongnam-do 31151 South Korea

**Keywords:** Breast cancer, Prognosis, Fluorodeoxyglucose F-18, Positron emission tomography, Bone marrow

## Abstract

**Background:**

The objective of this study was to investigate the prognostic value of 2-Deoxy-2-[^18^F]fluoro-D-glucose ([^18^F]FDG) uptake of bone marrow (BM) and metabolic parameters of primary tumor on positron emission tomography/computed tomography (PET/CT) for predicting distant recurrence in patients with breast cancer.

**Methods:**

Pretreatment [^18^F]FDG PET/CT images of 345 breast cancer patients were retrospectively evaluated. Maximum standardized uptake value, metabolic tumor volume, and total lesion glycolysis (TLG) of primary breast cancer and bone marrow-to-liver uptake ratio (BLR) on PET/CT were measured. A Cox proportional hazard regression model was used to evaluate the prognostic potential of parameters for predicting recurrence-free survival (RFS) and distant RFS. For Kaplan-Meier analysis, the specific cutoff values pf BLR and TLG were determined by the maximal chi-square method.

**Results:**

The median follow-up duration of the enrolled patients was 48.7 months, and during follow-up, 36 patients (10.4%) experienced the cancer recurrence. BLR was significantly correlated with T stage, serum inflammatory markers, and recurrence pattern (*p* < 0.05). Patients with high BLR and TLG showed worse RFS and distant RFS than those with low BLR and TLG. On multivariate analysis, BLR was significantly associated with both RFS and distant RFS after adjusting for T stage, estrogen receptor status, and TLG (*p* = 0.001 for both). Only 0.5% of patients with TLG < 9.64 g and BLR < 0.91 experienced distant recurrence. However, patients with TLG ≥ 9.64 g and BLR ≥ 0.91 had a distant recurrence rate of 40.7%.

**Conclusions:**

BLR on pretreatment [^18^F]FDG PET/CT were significant predictors for RFS and distant RFS in patients with breast cancer. By combining [^18^F]FDG uptake of BM and volumetric PET/CT index of breast cancer, the risk of distant recurrence could be stratified.

## Background

Recent evidences have shown that inflammation has a significant association with malignant disease [1, 2]. Nowadays, it is generally accepted that inflammatory microenvironment and immune response of the host plays significant roles in cancer proliferation, promotion, angiogenesis, and metastasis [1, 3]. For breast cancer, it has been found that multiple cytokines and chemokines produced by activated immune cells in the inflammatory microenvironment contribute to not only malignant transformation but also epithelial-to-mesenchymal transition that can lead to distant metastasis of cancer cells [4, 5]. In clinical settings, multiple serum-derived parameters such as neutrophil-to-lymphocyte ratio (NLR), platelet-to-lymphocyte ratio (PLR), and C-reactive protein have been used to estimate the degree of systemic inflammatory response to diseases [6–8]. In patients with breast cancer, the increase of the serum inflammatory markers has been linked to worse prognosis [7, 8]. Furthermore, the level of NLR in breast cancer patients has a significant association with the risk of distant recurrence after surgical resection [9, 10].

2-Deoxy-2-[^18^F]fluoro-D-glucose ([^18^F]FDG) positron emission tomography/computed tomography (PET/CT) has significant prognostic potential for predicting clinical outcomes in patients with breast cancer [11, 12]. Maximum [^18^F]FDG uptake of primary tumor has been the most commonly used predictive parameter of PET/CT; however, recent studies have reported that volumetric PET/CT parameters such as metabolic tumor volume (MTV) and total lesion glycolysis (TLG) have more significant association with survival than maximum [^18^F]FDG uptake in breast cancer [11–13]. In addition to the assessment of primary tumor features, a number of recent studies have demonstrated that the degree of systemic inflammatory response to malignancy can be evaluated with [^18^F]FDG PET/CT by measuring [^18^F]FDG uptake of bone marrow (BM) [14, 15]. Because [^18^F]FDG uptake of BM mainly reflects glucose metabolism of immune cells, increased [^18^F]FDG uptake of BM in patients with malignant diseases is considered to be due to BM activation caused by host inflammatory response [15, 16]. In various malignant diseases, [^18^F]FDG uptake of BM has shown significant correlations with serum inflammatory markers and clinical outcomes [14, 17–20]. However, in patients with breast cancer, only a single study have evaluated the clinical implication of [^18^F]FDG uptake of the reticuloendothelial system including BM and the spleen [21], and the prognostic potential of [^18^F]FDG uptake of BM for predicting distant recurrence has not been reported yet.

Thus, the main purpose of this study was to investigate the prognostic significance of [^18^F]FDG uptake of BM for predicting recurrence-free survival (RFS) and distant RFS in patients with breast cancer after curative surgical resection. We also evaluated whether the risk of distant recurrence could be stratified by combining PET/CT parameters of primary tumor and BM.

## Methods

### Patients

Electronic medical records of 382 female patients with histopathologically verified invasive breast cancer between February 2012 and October 2016 in our medical center were retrospectively reviewed. Among eligible patients, we finally enrolled 345 breast cancer patients who underwent pre-treatment staging [^18^F]FDG PET/CT and subsequently underwent curative surgical resection. The exclusion criteria were as follows: (1) those who were diagnosed with distant organ metastasis on staging imaging studies (*n* = 16); (2) those who were diagnosed with ductal carcinoma in situ (*n* = 3); (3) those who had any kind of treatment before [^18^F]FDG PET/CT scan (*n* = 1); (4) those who had any concurrent liver, hematologic, or infectious disease (*n* = 6); (5) those who had a previous history of another malignancy (*n* = 3); (6) those who had a history of granulocyte-macrophage colony-stimulating factor or erythropoietin injection within 6 months before [^18^F]FDG PET/CT scan (*n* = 2); and (7) those who were lost to follow-up within 2 years after the initial treatment without event (*n* = 6).

All clinico-pathological and survival data were obtained from medical records of patients. Prior to treatment, all patients underwent staging examinations including blood tests, breast ultrasonography, bone scintigraphy, breast magnetic resonance imaging, and [^18^F]FDG PET/CT. Based on results of staging imaging studies, clinical TNM stage was determined for each patient according to the 7th Edition of the American Joint Committee on Cancer staging system. Using blood cell count results of pretreatment blood tests, NLR and PLR were calculated for each patient. Estrogen receptor (ER), progesterone receptor (PR), human epidermal growth factor receptor 2 (HER2), and Ki67 expression status were obtained from histopathological records. ER- and PR-positive tumors were defined as tumors with 10% or more positively stained cells by immunohistochemistry. HER2-positive tumor was defined as tumor with a 3+ score on immunohistochemistry or tumor with gene amplification on fluorescence in situ hybridization. Tumor with 14% or more Ki67 expression by immunohistochemistry was defined as Ki67-positive tumor. After staging work-up, patients underwent curative surgical resection with or without neoadjuvant chemotherapy and/or adjuvant treatment according to staging and clinical condition of each patient. All enrolled patients were regularly followed up after treatments at intervals of every 3–6 months with blood tests and imaging examinations. Patients who experienced cancer recurrence during follow-up were categorized into two groups: patients with locoregional recurrence and patients with distant recurrence.

### [^18^F]FDG PET/CT scan

All patients had fasted for at least 6 h before [^18^F]FDG PET/CT scans. Approximately 4.07 MBq/kg of [^18^F]FDG was intravenously injected 1 h before PET/CT scans after confirmation of blood glucose level of less than 150 mg/dL. All [^18^F]FDG PET/CT scans were performed from the skull base to the proximal thigh in a supine position using a dedicated PET/CT scanner (Biograph mCT 128 scanner, Siemens Healthcare, Knoxville, TN, USA). A CT scan for attenuation correction and anatomical information was performed at 100 mA and 120 kV_p_ without contrast enhancement. A PET scan was performed at 1.5 min per bed position for 5–7 positions using three-dimensional acquisition mode. PET images were reconstructed using point-spread-function modeling and time-of-flight reconstruction (2 iterations and 21 subsets) with attenuation correction.

### [^18^F]FDG PET/CT image analysis

**Fig. 1:**
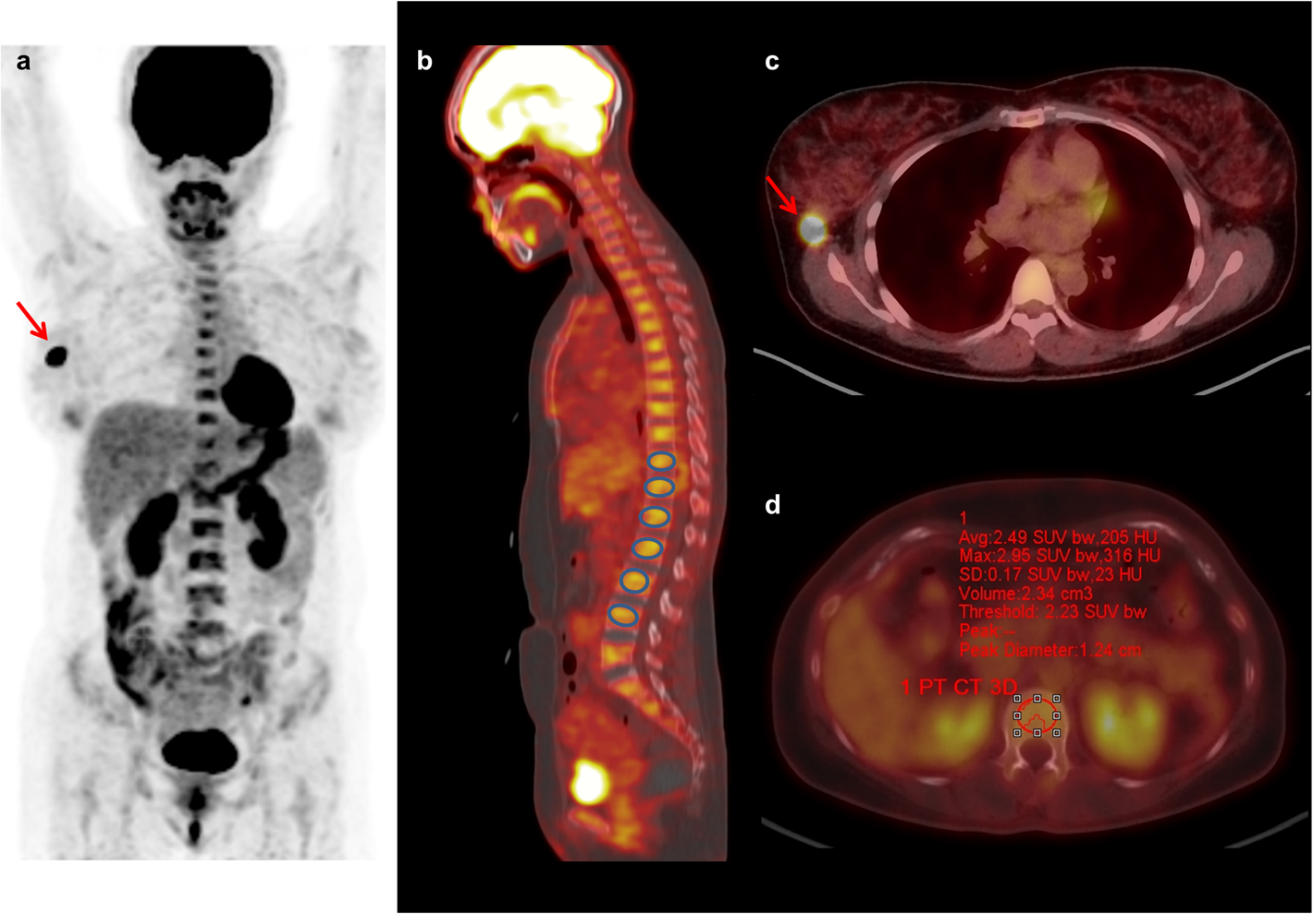
Maximum intensity projection (**a**), fused coronal PET/CT (**b**), and fused transaxial PET/CT (**c**, **d**), images of a 41-year-old woman with invasive breast cancer. Primary breast cancer lesion (arrow) showed intensely increased [^18^F]FDG uptake with maximum SUV of 17.30, MTV of 4.72 cm^3^, and TLG of 28.00 g (**a**, **c**). Six spheroid-shaped VOIs were manually drawn over the vertebral body of thoracic and lumbar spines (**b**). An isocontour using a cutoff SUV of 75% of the maximum SUV of VOI was automatically produced within each VOI of the vertebral body, and mean SUV of voxels within the isocontour was measured, showing mean SUV of L1 spine of 2.49 (**d**). BM SUV and BLR of the patient were 2.15 and 1.15, respectively. The patient was diagnosed with T2N0 stage and underwent curative surgical resection of the cancer lesion. Pulmonary metastases were found 21.9 months after the operation

[^18^F]FDG PET/CT images of all enrolled patients were retrospectively evaluated by two nuclear medicine physicians blinded to clinico-pathological and survival results. At first, metabolic parameters of primary breast cancer lesions were measured. A spheroid-shaped volume of interest (VOI) was drawn over the primary cancer lesion including the entire cancer lesion in axial, sagittal, and coronary planes. The maximum standardized uptake value (SUV) of primary breast cancer was measured. Within the VOI of primary breast cancer, voxels with SUV of 2.50 or greater were automatically selected. The total volume of those voxels within the VOI defined as MTV of primary tumor was measured, and the mean SUV of voxels was calculated. TLG of primary breast cancer was defined as multiplying MTV with mean SUV. Afterwards, two PET/CT parameters of BM, mean [^18^F]FDG uptake of BM (BM SUV) and bone marrow-to-normal liver uptake ratio (BLR), were measured according to methods described in previous studies [14, 17, 19]. A spheroid-shaped VOI was manually drawn over the vertebral body of six vertebrae of thoracic and lumbar spines (Fig. 1). Vertebrae that showed severe osteoarthritic change, compression fracture, benign bone tumor, or post-operative change of spinal surgery were excluded from the measurement. An isocontour using a cutoff SUV of 75% of the maximum SUV of VOI was automatically produced within each VOI of the vertebral body (Fig. 1d). Mean SUV of voxels within the isocontour was measured and defined as SUV of the vertebral body. The average value of SUV of six vertebral bodies selected was calculated and defined as BM SUV. A spheroid-shaped 3-cm-sized VOI were drawn over the liver, right lobe, and mean SUV of the VOI was defined as mean SUV of normal liver tissue. Using BM SUV and mean SUV of the normal liver, BLR was calculated for each patient.

### Statistical analysis

Kruskal-Wallis test and Student *t* test were used to compare values of [^18^F]FDG PET/CT parameters between patient groups. To evaluate relationships of BM SUV and BLR with tumor size, serum inflammatory markers, and PET/CT parameters of primary breast cancer, Spearman rank correlation coefficients were calculated after performing normality test. To assess the prognostic values of [^18^F]FDG PET/CT parameters of primary tumor and BM and clinico-pathological factors for predicting RFS and distant RFS, univariate and multivariate analyses were performed using Cox proportional hazards regression with calculating the Harrell concordance index (C-index). Survival time was defined as the time from the initial treatment to the day of the detection of cancer recurrence (RFS) or distant recurrence (distant RFS). For patients without cancer recurrence, follow-up was censored at the day of the last visit at our medical center. For both univariate and multivariate survival analyses, Bonferroni correction was applied to adjust for multiple testing. In the multivariate analysis of [^18^F]FDG uptake of BM, considering the number of variables as compared with the number of events [22], we made five different models with eight different covariates that showed statistical significance in the univariate analysis. The significance of the predictive value of BM [^18^F]FDG uptake for RFS and distant RFS was assessed in these five models after adjusting for the eight covariates. For estimating survival curves for [^18^F]FDG PET/CT parameters, the specific cutoff values were determined using the maximal chi-square method and the Kaplan-Meier method was used to calculate cumulative RFS and distant RFS. Fisher’s exact test was performed to evaluate differences of distant recurrence rates according to PET/CT parameters. All statistical analyses were performed using MedCalc Statistical Software version 18.11.3 (MedCalc Software bvba. Ostend. Belgium) and R software version 3.5.3 (The R Foundation for Statistical Computing, Vienna, Austria). Statistical significance was considered when *p* value was less than 0.05.

## Results

### Patient characteristics

Clinico-pathological characteristics and [^18^F]FDG PET/CT parameters of enrolled female breast cancer patients (*n* = 345) are summarized in Table 1 (Supplementary Fig. 1). Among these 345 enrolled patients, 122 (35.4%) had regional lymph node metastasis on staging work-up and 42 (12.2%) had triple-negative breast cancer. The interval between [^18^F]FDG PET/CT and initial treatment was within 2 weeks for all patients. Neoadjuvant chemotherapy was performed for 48 patients (13.9%). After curative surgical resection of breast cancer, adjuvant treatments were performed for 340 patients (98.6%). The median follow-up duration of enrolled patients was 48.7 months (range, 6.1–74.7 months). During follow-up, cancer recurrence was found in 36 patients (10.4%), and among them, distant recurrence was found in 20 patients (5.8%). The most common site of distant recurrence was the lung, followed by the bone, liver, adrenal gland, and brain.

**Table 1:** Characteristics of 345 patients

Characteristics		Number (%)	Median (range)
Age (years)			51 (30–85)
Menopausal status	Premenopausal	146 (42.3%)	
	Postmenopausal	199 (57.7%)	
Histopathology	Invasive ductal carcinoma	307 (89.0%)	
	Invasive lobular carcinoma	38 (11.0%)	
T stage	T1	158 (45.8%)	
	T2	151 (43.8%)	
	T3	24 (7.0%)	
	T4	12 (3.5%)	
N stage	N0	223 (64.6%)	
	N1	68 (19.7%)	
	N2	29 (8.4%)	
	N3	25 (7.3%)	
Tumor size (cm)			2.0 (0.4–15.0)
Histologic grade	Grade 1	82 (23.8%)	
	Grade 2	173 (50.1%)	
	Grade 3	90 (26.1%)	
Estrogen receptor status	Positive	258 (74.8%)	
	Negative	87 (25.2%)	
Progesterone receptor status	Positive	212 (61.4%)	
	Negative	133 (38.6%)	
HER2 status	Positive	176 (51.0%)	
	Negative	169 (49.0%)	
Ki67 expression status	Positive (≥ 14%)	230 (66.7%)	
	Negative (< 14%)	115 (33.3%)	
WBC (× 10^12^ cells/L)			6.36 (2.47–17.56)
NLR			1.66 (0.29–11.36)
PLR			120.20 (20.63–600.00)
Maximum SUV of primary tumor			4.10 (1.10–37.90)
MTV of primary tumor (cm^3^)			1.14 (0.0–528.58)
TLG of primary tumor (g)			3.45 (0.0–3311.99)
BM SUV			1.61 (0.62–2.73)
BLR			0.76 (0.35–1.54)
Neoadjuvant chemotherapy	Yes	48 (13.9%)	
	No	297 (86.1%)	
Adjuvant treatment	CTx+RTx+HTx	165 (47.8%)	
	RTx+HTx	100 (29.0%)	
	CTx+HTx	19 (5.5%)	
	CTx+RTx	5 (1.4%)	
	HTx	28 (8.1%)	
	CTx	20 (5.8%)	
	RTx	3 (0.9%)	
	No	5 (1.4%)	

*HER2* human epidermal growth factor receptor 2, *WBC* white blood cell, *NLR* neutrophil-to-lymphocyte ratio, *PLR* platelet-to-lymphocyte ratio, *SUV* standardized uptake value, *MTV* metabolic tumor volume, *TLG* total lesion glycolysis, *BM* bone marrow, *BLR* bone marrow-to-liver uptake ratio, *CTx* chemotherapy, *RTx* radiotherapy, *HTx* hormonal therapy

### [^18^F]FDG PET/CT parameters of primary breast cancer

Among enrolled patients, MTV and TLG were set to be 0.0 cm^3^ and 0.0 g, respectively, in 111 patients (32.2%), because values of maximum SUV of primary breast cancer were less than 2.50. Kruskal-Wallis test revealed significant differences of maximum SUV, MTV, and TLG among patients with distant recurrence, locoregional recurrence, and no recurrence (Table 2; all *p* < 0.0001). Post hoc analysis showed that patients with distant recurrence and locoregional recurrence had significantly higher values of maximum SUV, MTV, and TLG than those without recurrence (*p* < 0.05). However, there were no significant differences in all three PET/CT parameters between patients with distant recurrence and those with locoregional recurrence (*p* > 0.05).

**Table 2:** Relationship of recurrence pattern with [^18^F]FDG PET/CT parameters of primary breast cancer and BM

		No recurrence (*n* = 309)	Locoregional recurrence (*n* = 16)	Distant recurrence (*n* = 20)	*p* value
Maximum SUV	Median	3.69	7.21	9.20	< 0.001*
	Range	0.90–37.90	2.36–17.96	1.95–17.47	
MTV	Median	0.78	5.26	10.00	< 0.001*
	Range	0.0–234.50	0.0–235.30	0.0–528.58	
TLG	Median	2.33	25.90	42.43	< 0.001*
	Range	0.0–1674.52	0.0–1682.09	0.0–3311.99	
BM SUV	Median	1.60	1.64	1.75	0.090
	Range	0.62–2.73	1.27–2.45	1.11–1.96	
BLR	Median	0.75	0.80	0.98	< 0.001^†^
	Range	0.35–1.54	0.51–1.16	0.50–1.33	

*SUV* standardized uptake value, *MTV* metabolic tumor volume, *TLG* total lesion glycolysis, *BM* bone marrow, *BLR* bone marrow-to-liver uptake ratio

*On post hoc analysis, patients with locoregional and distant recurrence had significantly higher values than those with no recurrence (*p* < 0.05). However, no significant difference was shown between patients with locoregional recurrence and those with distant recurrence (*p* > 0.05)

†On post hoc analysis, patients with distant recurrence showed significantly higher value than those with no recurrence and locoregional recurrence (*p* < 0.05). However, no significant difference was shown between patients with no recurrence and those with locoregional recurrence (*p* > 0.05)

### [^18^F]FDG PET/CT parameters of BM

**Fig. 2:**
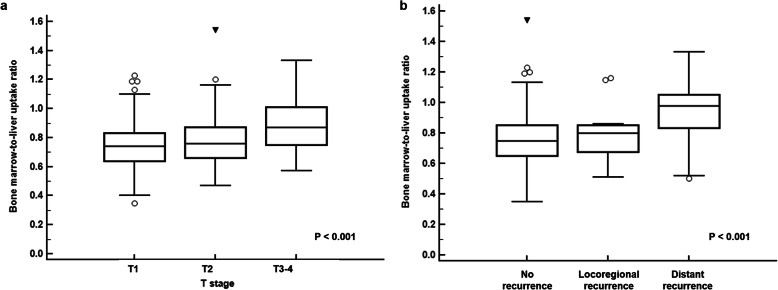
Bone marrow-to-liver uptake ratio according to T stage (**a**) and recurrence pattern (**b**)

Of all patients, 33 (9.6%) showed [^18^F]FDG uptake of BM that was higher than [^18^F]FDG uptake of the normal liver tissue (BLR > 1.0; Supplementary Fig. 1). Relationships of [^18^F]FDG uptake of BM with clinico-pathological factors, serum inflammatory markers, and [^18^F]FDG PET/CT parameters of primary breast cancer were evaluated. For clinico-pathological factors, significant differences of BM SUV and BLR were shown according to the T stage (*p* = 0.039 for BM SUV and *p* < 0.001 for BLR; Fig. 2a). On post hoc analysis, patients with T3–4 stages [median, 1.72 (range, 0.73–2.50) for BM SUV; median, 0.87 (range, 0.57–1.33) for BLR] had significantly higher values of BM SUV and BLR than those with T1 stage [median, 1.60 (range, 0.62–2.41) for BM SUV; median, 0.74 (range, 0.35–1.23) for BLR] and T2 stage [median, 1.61 (range, 0.80–2.73) for BM SUV; median, 0.76 (range, 0.47–1.54) for BLR] (*p* < 0.05 for both BM SUV and BLR). In contrast, neither BM SUV nor BLR showed significant association with N stage, histologic grade, tumor size, status of ER, PR, HER2, or Ki67 expression (*p* > 0.05). In correlation analysis with serum inflammatory markers, BM SUV and BLR showed significant positive correlations with white blood cell counts (*p* = 0.019, *r* = 0.126 for BM SUV; *p* = 0.021, *r* = 0.125 for BLR), NLR (*p* = 0.001, *r* = 0.207 for BM SUV; *p* < 0.001, *r* = 0.284 for BLR), and PLR (*p* = 0.004, *r* = 0.185 for BM SUV; *p* < 0.001, *r* = 0.273 for BLR). There were no significant correlations of BM SUV or BLR with hemoglobin level (*p* > 0.05).

In correlation analysis with [^18^F]FDG PET/CT parameters of primary tumor, only maximum SUV of primary cancer showed significant but weakly positive correlations with BM SUV (*p* = 0.036, *r* = 0.113) and BLR (*p* = 0.041, *r* = 0.108). BM [^18^F]FDG uptake showed no significant correlation with MTV or TLG (*p* > 0.05). Relationships of recurrence pattern with PET/CT parameters of BM were also assessed (Table 2). On the Kruskal-Wallis test, significant differences of BLR were found among patients with distant recurrence, locoregional recurrence, and no recurrence (*p* < 0.001; Fig. 2b). However, there was no significant relationship between BM SUV and recurrence pattern (*p* = 0.090). On post hoc analysis, patients with distant recurrence had significantly higher values of BLR than those without recurrence and those with locoregional recurrence (*p* < 0.05). There was no significant difference of BLR between patients without recurrence and those with locoregional recurrence (*p* > 0.05).

### Survival analysis

The prognostic potential of [^18^F]FDG PET/CT parameters of primary cancer and BM for predicting RFS and distant RFS was assessed using univariate Cox regression analysis along with clinico-pathological factors (Table 3). Bonferroni correction for multiple testing was performed for the univariate analysis, and a *p* value of < 0.003 was considered as statistically significant. Of PET/CT parameters, MTV, TLG, and BLR were significantly associated with RFS and distant RFS in univariate survival analysis. Maximum SUV showed a significant association with only RFS. Along with PET/CT parameters, T stage, ER status, and triple-negative breast cancer were significant prognostic factors for predicting both RFS and distant RFS. N stage and PR status were significant predictors for only RFS, and PLR was a significant predictor for only distant RFS.

**Table 3:** Univariate analysis for recurrence-free survival and distant recurrence-free survival

Variables	Recurrence-free survival		Distant recurrence-free survival	
	*p* value*	HR (95% CI)	*p* value*	HR (95% CI)
Age (1-year increase)	0.517	1.01 (0.98–1.04)	0.836	0.98 (0.94–1.02)
Menopausal status (pre vs. post)	0.998	1.01 (0.52–1.94)	0.224	0.58 (0.24–1.40)
T stage				
T1 stage vs. T2 stage	< 0.001	8.43 (2.51–28.31)	0.034	5.24 (1.13–24.30)
T1 stage vs. T3–4 stage	< 0.001	25.08 (7.06–89.10)	< 0.001	26.04 (5.62–120.78)
N stage (N0 vs. N1–3)	0.001	2.97 (1.53–5.77)	0.044	2.48 (1.03–5.99)
Histologic grade				
Grade 1 vs. grade 2	0.419	1.59 (0.52–4.87)	0.403	1.94 (0.41–9.12)
Grade 1 vs. grade 3	0.003	5.03 (1.71–14.81)	0.036	5.07 (1.11–23.20)
ER status (positive vs. negative)	< 0.001	3.93 (2.04–7.57)	< 0.001	5.07 (2.07–12.43)
PR status (positive vs. negative)	< 0.001	4.82 (2.32–10.01)	0.031	2.68 (1.09–6.57)
HER2 status (positive vs. negative)	0.612	1.19 (0.61–2.29)	0.872	0.93 (0.39–2.24)
Ki67 index (negative vs. positive)	0.057	5.77 (0.98–18.82)	0.090	2.89 (0.85–9.88)
Triple negative tumor (no vs. yes)	< 0.001	3.70 (1.77–7.73)	< 0.001	5.07 (2.00–12.84)
NLR (1.00 increase)	0.221	1.11 (0.94–1.33)	0.052	1.21 (0.99–1.46)
PLR (1.0 increase)	0.007	1.01 (1.00–1.01)	0.001	1.01 (1.00–1.01)
Maximum SUV (1.00 increase)	< 0.001	1.07 (1.03–1.11)	0.032	1.06 (1.01–1.12)
MTV (1.00 cm^3^ increase)	< 0.001	1.01 (1.01–1.01)	< 0.001	1.01 (1.01–1.02)
TLG (1.00 g increase)	< 0.001	1.00 (1.00–1.01)	< 0.001	1.01 (1.00–1.01)
BM SUV (1.00 increase)	0.041	1.86 (1.03–4.72)	0.048	2.32 (1.02–5.99)
BLR (1.00 increase)	< 0.001	22.34 (4.70–46.23)	< 0.001	29.76 (11.05–69.34)

*ER* estrogen receptor, *PR* progesterone receptor, *HER2* human epidermal growth factor receptor 2, *NLR* neutrophil-to-lymphocyte ratio, *PLR* platelet-to-lymphocyte ratio, *SUV* standardized uptake value, *MTV* metabolic tumor volume, *TLG* total lesion glycolysis, *BM* bone marrow, *BLR* bone marrow-to-liver uptake ratio, *HR* hazard ratio, *CI* confidence interval

*Statistically significant for *p* value < 0.003

Multivariate survival analysis for BLR was performed with the addition of eight different covariates (T stage, N stage, ER status, PR status, triple-negative tumor, PLR, maximum SUV, and TLG) that showed statistical significance in the univariate analysis (Table 4). Bonferroni correction for multiple testing was performed for each model in multivariate analysis. For volumetric PET/CT parameters of primary cancer, only TLG was included in the multivariate analysis, because MTV and TLG were significantly correlated with each other (*p* < 0.001; *r* = 0.961). In multivariate models, BLR was a significant predictor for both RFS and distant RFS after adjustment for tumor stage (model 1), hormone receptor status (model 2), serum inflammatory marker (model 3), and PET/CT parameters of primary tumor (model 4). In the final model (model 5) which included the covariates that showed statistical significance in the multivariate models (model 1–4), BLR remained a significant predictor for both RFS and distant RFS (*p* = 0.001 for both), along with T stage and ER status.

**Table 4:** Multivariate analysis for recurrence-free survival and distant recurrence-free survival

Model	Variables	Recurrence-free survival		Distant recurrent-free survival	
		*p* value	HR (95% CI)	*p* value	HR (95% CI)
Model 1*	T2 stage (vs. T1 stage)	0.002	6.72 (1.97–22.90)	0.078	4.06 (0.85–19.29)
	T3–4 stage (vs. T1 stage)	< 0.001	14.30 (3.65–56.12)	0.002	15.96 (2.90–87.91)
	N1–3 stage (vs. N0 stage)	0.187	1.66 (0.78–3.50)	0.849	1.11 (0.39–3.17)
	BLR (1.0 increase)	0.006	11.04 (1.96–62.15)	0.001	34.16 (4.05–288.23)
Model 2**	Negative ER (vs. positive)	0.002	4.55 (1.73–12.00)	0.003	8.71 (2.58–64.19)
	Negative PR (vs. positive)	0.537	1.36 (0.51–3.58)	0.518	0.52 (0.07–3.81)
	Triple negative tumor	0.524	1.34 (0.54–3.30)	0.273	1.92 (0.60–6.18)
	BLR (1.0 increase)	< 0.001	51.41 (9.35–282.74)	< 0.001	162.31 (20.73–1271.00)
Model 3***	PLR (1.0 increase)	0.048	1.00 (1.00–1.01)	0.030	1.01 (1.00–1.01)
	BLR (1.0 increase)	< 0.001	18.08 (3.52–92.80)	< 0.001	61.13 (8.58–435.36)
Model 4*	Maximum SUV (1.00 increase)	0.025	1.05 (1.01–1.10)	0.183	1.05 (0.98–1.12)
	TLG (1.00 g increase)	< 0.001	1.01 (1.00–1.01)	0.010	1.01 (1.00–1.01)
	BLR (1.0 increase)	< 0.001	23.16 (4.51–119.06)	< 0.001	62.59 (8.76–447.36)
Model 5**	T2 stage (vs. T1 stage)	0.004	6.16 (1.82–20.88)	0.136	3.25 (0.69–15.30)
	T3–4 stage (vs. T1 stage)	< 0.001	11.09 (2.90–42.32)	0.007	9.49 (1.85–48.68)
	Negative ER (vs. positive)	0.003	2.90 (1.44–5.86)	0.001	4.88 (1.86–12.82)
	TLG (1.00 g increase)	0.056	1.00 (1.00–1.01)	0.484	1.00 (1.00–1.00)
	BLR (1.0 increase)	0.001	16.38 (2.94–91.14)	0.001	80.45 (8.87–729.86)

*BLR* bone marrow-to-liver uptake ratio, *ER* estrogen receptor, *PR* progesterone receptor, *PLR* platelet-to-lymphocyte ratio, *SUV* standardized uptake value, *TLG* total lesion glycolysis, *HR* hazard ratio, *CI* confidence interval

*Statistically significant for *p* value < 0.017

**Statistically significant for *p* value < 0.013

***Statistically significant for *p* value < 0.025

**Fig. 3:**
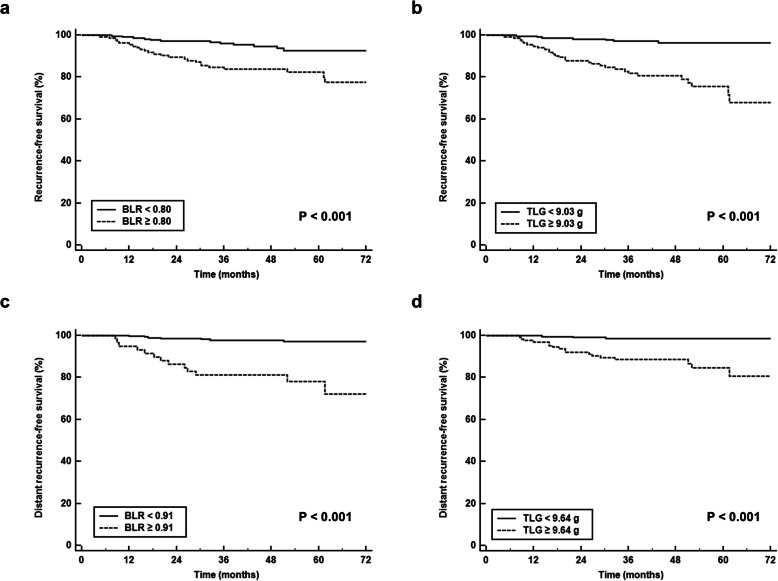
Recurrence-free survival stratified by bone marrow-to-liver uptake ratio (BLR) (**a**) and total lesion glycolysis (TLG) of primary breast cancer (**b**). Distant recurrence-free survival stratified by BLR (**c**) and TLG of primary breast cancer (**d**)

For Kaplan-Meier analysis, BLR and TLG were categorized into two groups according to the specific cutoff values determined by the maximal chi-square method (BLR: 0.80 for RFS and 0.91 for distant RFS; TLG: 9.03 g for RFS and 9.64 g for distant RFS). In Kaplan-Meier analysis, patients with high BLR and TLG showed significantly worse RFS and distant RFS than those with low BLR and TLG, respectively (*p* < 0.001 for all, Fig. 3). On the Harrell’s C statistical analysis, both BLR and TLG showed great discriminative ability in predicting distant RFS (BLR: C-index, 0.762; 95% confidence interval, 0.633–0.891; TLG: C-index, 0.760; 95% confidence interval, 0.644–0.877) as well as in predicting RFS (BLR: C-index, 0.699; 95% confidence interval, 0.626–0.771; TLG: C-index 0.746; 95% confidence interval, 0.660–0.832).

### Distant recurrence rate according to TLG and BLR

To further enhance the predictive value of [^18^F]FDG PET/CT, we evaluated distant recurrence rates according to the combination of TLG and BLR (Table 5). For 27 patients who showed TLG ≥ 9.64 g and BLR ≥ 0.91 on PET/CT, the distant recurrence rate was high at 40.7%. On the other hand, only one patient among 140 patients (0.5%) with low TLG and BLR values experienced distant recurrence. Significant differences of distant recurrence rates were found according to cutoff values of TLG and BLR (*p* < 0.05; Table 5).

**Table 5:** Distant recurrence rate according to the combination of TLG and BLR

		TLG		
		< 9.64 g	≥ 9.64 g	*p* value
BLR	< 0.91	1/184 (0.5%)	6/101 (5.9%)	0.005
	≥ 0.91	2/33 (6.1%)	11/27 (40.7%)	0.002
	*p* value	0.013	< 0.001	

*TLG* total lesion glycolysis, *BLR* bone marrow-to-liver uptake ratio

## Discussion

The present study found that BLR measured on pretreatment [^18^F]FDG PET/CT was independently associated with distant RFS as well as RFS in patients with breast cancer. A previous study has reported that only mild [^18^F]FDG uptake is observed in the BM of normal healthy subjects, showing mean BM SUV and BLR of 1.31 ± 0.21 and 0.60 ± 0.10, respectively [23]. On the other hand, physicians have encountered an increase of [^18^F]FDG uptake of BM in a significant portion of patients with malignant diseases, even in those without concurrent infectious or hematologic diseases [23, 24]. Previous studies reported that BM SUV and BLR have significant positive correlations with white blood cell counts, serum C-reactive protein level, NLR, PLR, and transforming growth factor (TGF)-beta [14, 16–19, 24–26]. A number of previous studies have demonstrated that PET parameters of BM were significant predictors for survival in various kinds of cancers including small cell lung cancer, non-small cell lung cancer, gastric cancer, cervical cancer, colorectal cancer, lymphoma, malignant pleural mesothelioma, melanoma, and head and neck cancer [14, 17–19, 23–25, 27–32]. Based on these results, [^18^F]FDG uptake of BM has been suggested as an imaging biomarker for evaluating the degree of systemic inflammatory response and predicting cancer progression [14, 18, 19].

The results of our study showed significant positive correlations of [^18^F]FDG uptake of BM with serum inflammatory markers. In addition, BLR was determined to be a significant predictor for RFS, indicating that [^18^F]FDG uptake of BM could also serve as PET/CT parameters for estimating inflammatory response of the host in breast cancer. Furthermore, there were significant positive correlations of [^18^F]FDG uptake of BM with T stage and maximum SUV of breast cancer, suggesting enhanced systemic inflammatory response in patients with aggressive tumors, consistent with results of previous studies [17, 18, 25, 33]. Considering that epithelial-mesenchymal transition of breast cancer cells is promoted by neutrophils and macrophages via multiple secretory factors such as S100A8/A9 proteins, tumor necrosis factor-alpha, and TGF-beta [5, 34, 35], we hypothesized that the risk of distant recurrence could be also associated with the degree of [^18^F]FDG uptake of BM. In accordance with our hypothesis, our results demonstrated that patients with distant recurrence had significantly higher BLR than those without recurrence and with locoregional recurrence. Furthermore, BLR remained as a significant predictor for distant RFS even after adjusting the tumor stage, ER status, and TLG. Recently, several studies have attempted to treat breast cancer by modulating immune cells and inflammatory condition in tumor microenvironment [36, 37]. For future clinical trials that evaluate the effect of such a treatment, patients with high BLR might be good candidates.

In contrast with maximum SUV, MTV and TLG can reflect the extent of metabolically active tumor burden [38, 39]. They have shown superior prognostic values for predicting clinical outcomes in various malignant diseases [39, 40]. In previous studies on breast cancer patients, although volumetric parameters of [^18^F]FDG PET/CT cannot be used to differentiate hormone receptor status of cancer, TLG was significantly associated with aggressive tumor features and the presence of systemic metastasis [11–13]. In addition, TLG was a significant predictor for disease-free survival and overall survival in breast cancer patients [11–13]. Therefore, it was expected that TLG had significant associations with both RFS and distant RFS in the present study. Moreover, in the multivariate model with maximum SUV, TLG, and BLR for predicting distant recurrence, only TLG and BLR showed statistical significance.

Successful metastatic event of breast cancer cells is known to be governed by not only biological characteristics of cancer cells, but also complex interactions between cancer cells and normal host cells including immune cells based on the “seed and soil theory” [41, 42]. According to the results of the present study, the risk of distant recurrence of breast cancer could be further stratified by combining TLG of breast cancer and BLR. More than 40% of patients with high values of TLG and BLR experienced distant recurrence, and both BLR and TLG showed good discriminative ability in predicting distant RFS as well as RFS. These results imply that both tumor aggressiveness expressed as TLG (seed) and inflammatory response of the host expressed as BLR (soil) can be quantitatively estimated by a single [^18^F]FDG PET/CT scan before treatment of breast cancer and that combination of those PET parameters can be used to predict the risk of distant recurrence. When recurrence is suspected in patients who show high TLG of breast cancer and BLR on pretreatment [^18^F]FDG PET/CT, systemic evaluation should be performed for the detection of hidden distant metastasis.

In the literature, only one retrospective study has assessed the prognostic potential of [^18^F]FDG uptake of BM in 153 patients with invasive ductal carcinoma of the breast [21]. In that study, maximum SUV of primary tumor and mean SUV of the liver, spleen, and BM were measured and relationships of the PET/CT parameters with recurrence after curative surgical resection were evaluated. Similar to the results of our study, that study revealed significant associations between BM SUV and maximum SUV of primary tumor and between BM SUV and disease-free survival on univariate analysis. However, on multivariate survival analysis, only mean SUV of the spleen was included because mean SUV of the spleen showed the most significant predictive value among mean SUV of the liver, spleen, and BM. Different from that previous study, we calculated additional PET/CT parameter for BM, the BLR. BLR is a parameter which corrects BM SUV by mean [^18^F]FDG uptake of the liver to reduce inter-individual variation in BM SUV [16]. A number of previous studies have demonstrated that BLR has higher correlation coefficients with serum inflammatory markers than BM SUV and that it is a more preferable parameter than BM SUV on survival analysis [14, 17, 19, 20, 24]. In the present study, BLR also showed more significant associations with recurrence pattern, NLR, and PLR than BM SUV and was an independent prognostic factor for predicting survival. In future studies, the spleen-to-liver uptake ratio should be measured like BLR and the comparison of prognostic significance between BLR and spleen-to-liver uptake ratio is needed.

There are several limitations that remained to be addressed in the present study. First of all, this study was retrospectively performed with patients enrolled from a single center. Due to small numbers of events with relatively short follow-up durations for evaluating breast cancer recurrence, there might be limitation in interpreting the results of the study. Therefore, further studies with multi-center cohort and long-term follow-up are necessary to validate the results of the study. Second, because distant recurrence was diagnosed mainly by imaging examinations, the exact incidence and recurrence timing might be underestimated. Third, because MTV and TLG values of patients with maximum SUV of primary breast cancer < 2.50 were set to be 0.0 cm^3^ and 0.0 g, respectively, metabolic tumor burden in patients with low [^18^F]FDG uptake would be underestimated. Fourth, although there was no significant association between [^18^F]FDG uptake of BM and hemoglobin level, hematopoiesis of red blood cells might confound [^18^F]FDG uptake of BM [17, 21, 25]. Finally, because of the retrospective nature of the study, we could not assess the relationship of [^18^F]FDG uptake of BM with the other well-known prognostic factors for systemic inflammatory response such as the Glasgow prognostic score which has been found to be associated with poor prognosis in patients with malignant diseases including breast cancer [43, 44]. Therefore, further prospective studies with laboratory as well as histopathologic results are needed to elucidate the connection between BM metabolism on PET/CT and systemic inflammatory response.

## Conclusion

Increased [^18^F]FDG uptake of BM on pretreatment [^18^F]FDG PET/CT was observed in breast cancer patients with advanced T stage and distant recurrence. Patients with a high [^18^F]FDG uptake of BM showed worse survival than those with low values. BLR showed independent significant association with both RFS and distant RFS, and the risk of distant recurrence after curative surgical resection can be further stratified by combining TLG and BLR values. [^18^F]FDG PET/CT could provide metabolic information of both primary tumor and BM, which might help predict the risk of distant recurrence in patients with breast cancer.

## Supplementary information

**Additional file 1: Figure 1.** Histograms of maximum SUV (a), MTV (b), TLG (c), BM SUV (d), and BLR (e).

## Data Availability

The datasets used and/or analyzed during the current study are available from the corresponding author on reasonable request.

## Abbreviations

BLR: Bone marrow-to-liver uptake ratio
BM: Bone marrow
ER: Estrogen receptor
[^18^F]FDG: 2-Deoxy-2-[^18^F]fluoro-D-glucose
HER2: Human epidermal growth factor receptor 2
MTV: Metabolic tumor volume
NLR: Neutrophil-to-lymphocyte ratio
PET/CT: Positron emission tomography/computed tomography
PLR: Platelet-to-lymphocyte ratio
PR: Progesterone receptor
RFS: Recurrence-free survival
ROC: Receiver operating characteristic
SUV: Standardized uptake value
TGF: Transforming growth factor
TLG: Total lesion glycolysis
VOI: Volume of interest
