# Supplementary figures and images for: [18F]FDG uptake of bone marrow on PET/CT for predicting distant recurrence in breast cancer patients after surgical resection
Source: EJNMMI Res. 2020 Jun 30;10:72. doi: 10.1186/s13550-020-00660-y (PMC7326752; doi:10.1186/s13550-020-00660-y)

**Supplementary Fig. 1**. Histograms of maximum SUV (a), MTV (b), TLG (c), BM SUV (d), and BLR (e).


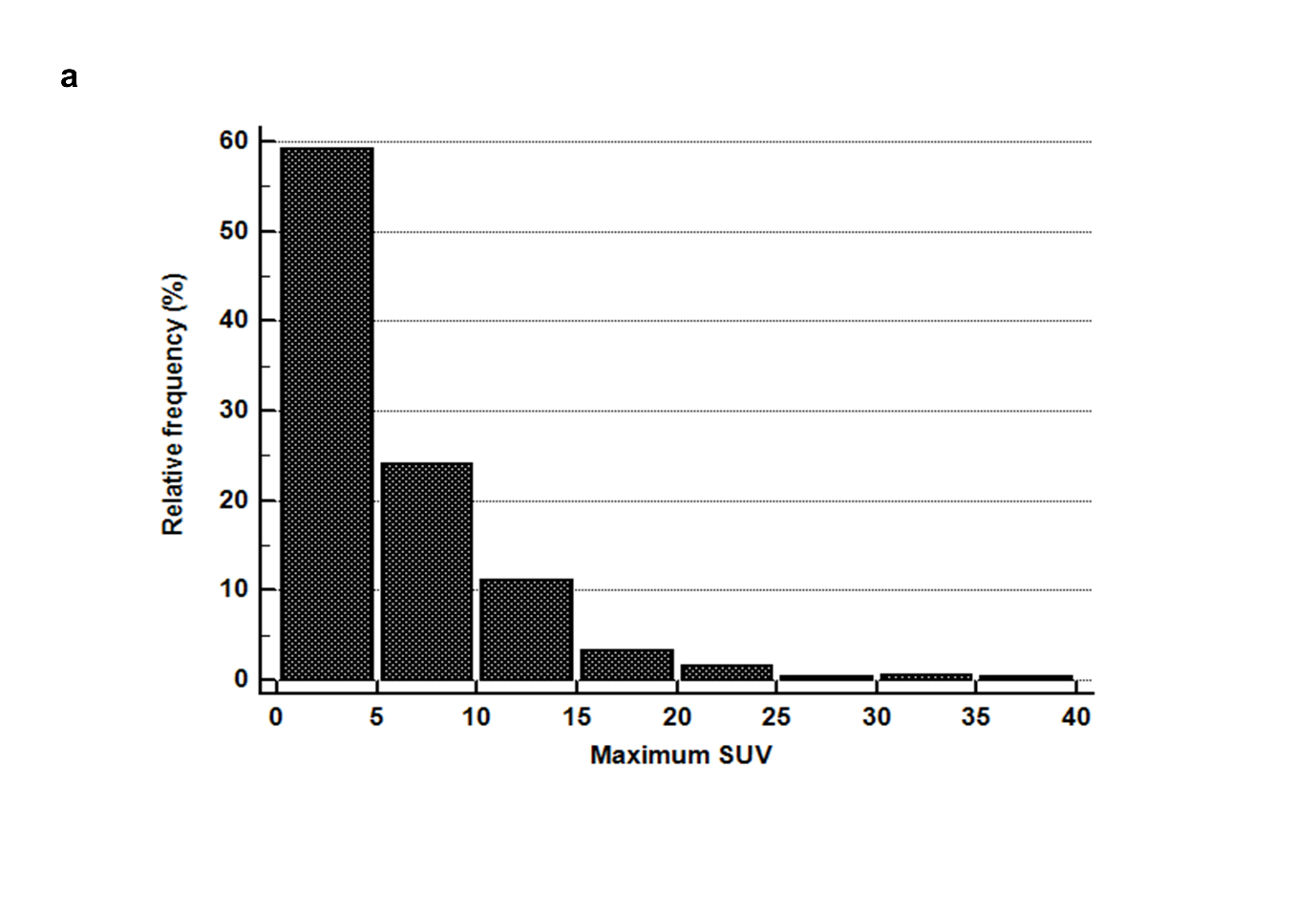


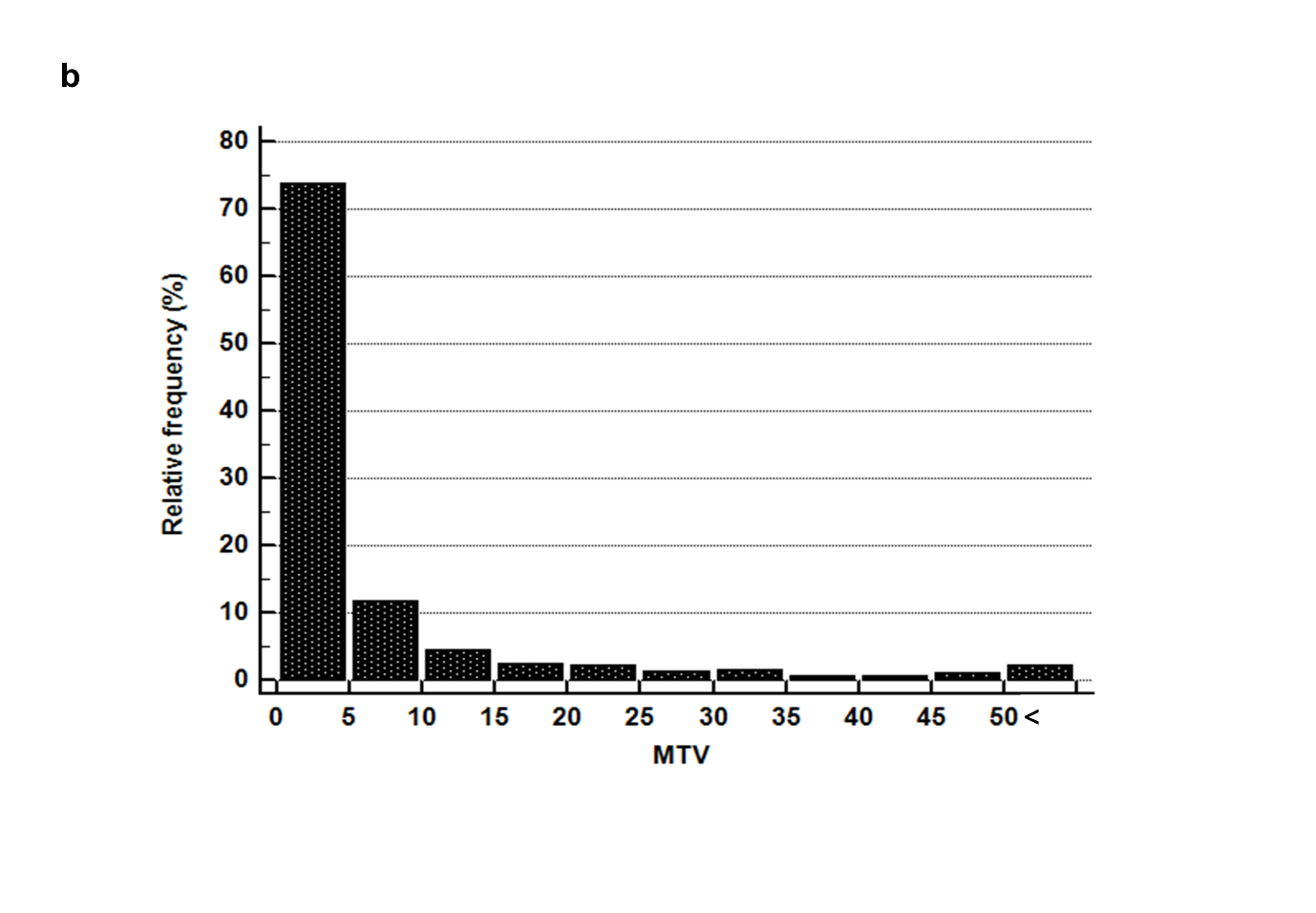


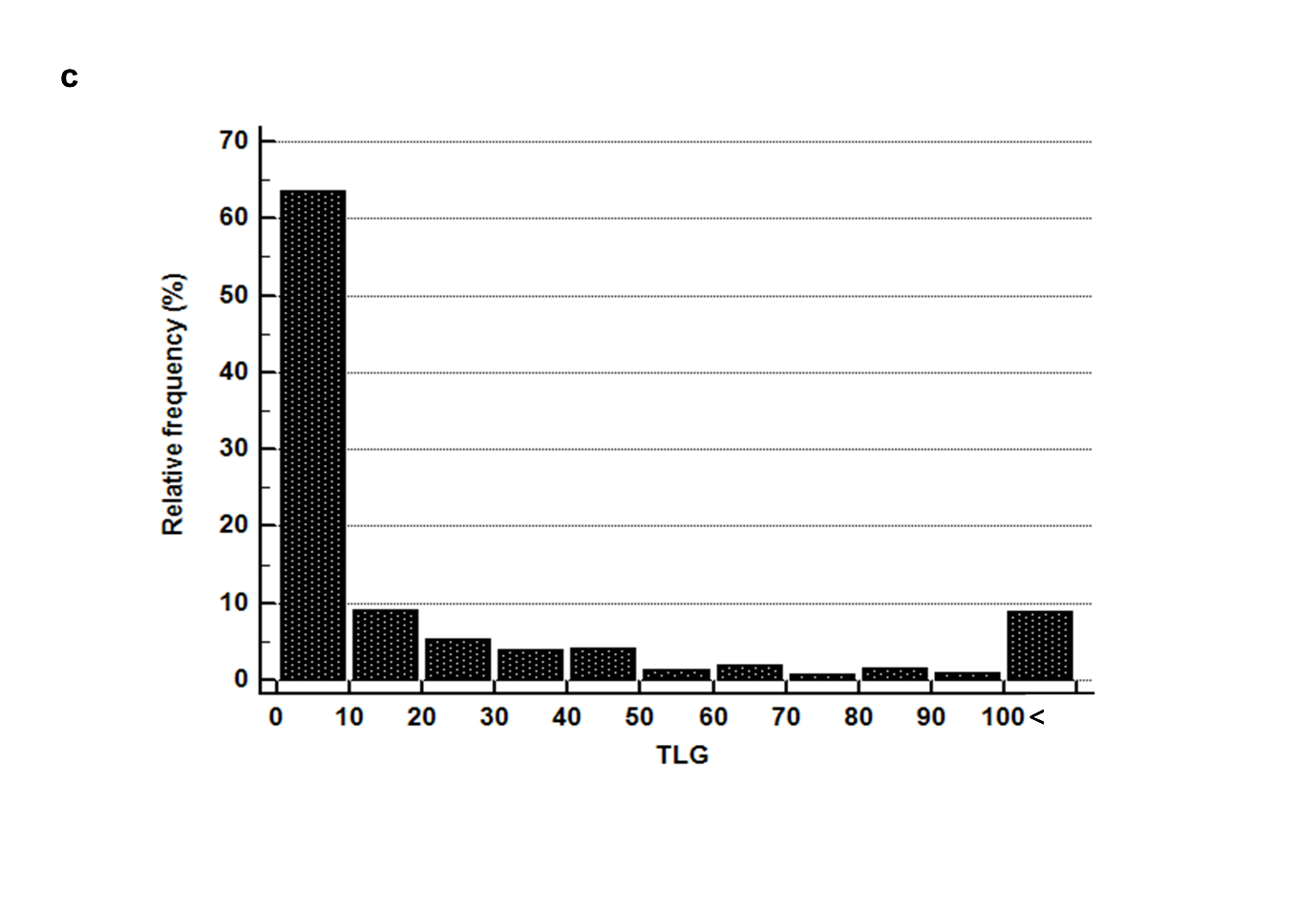


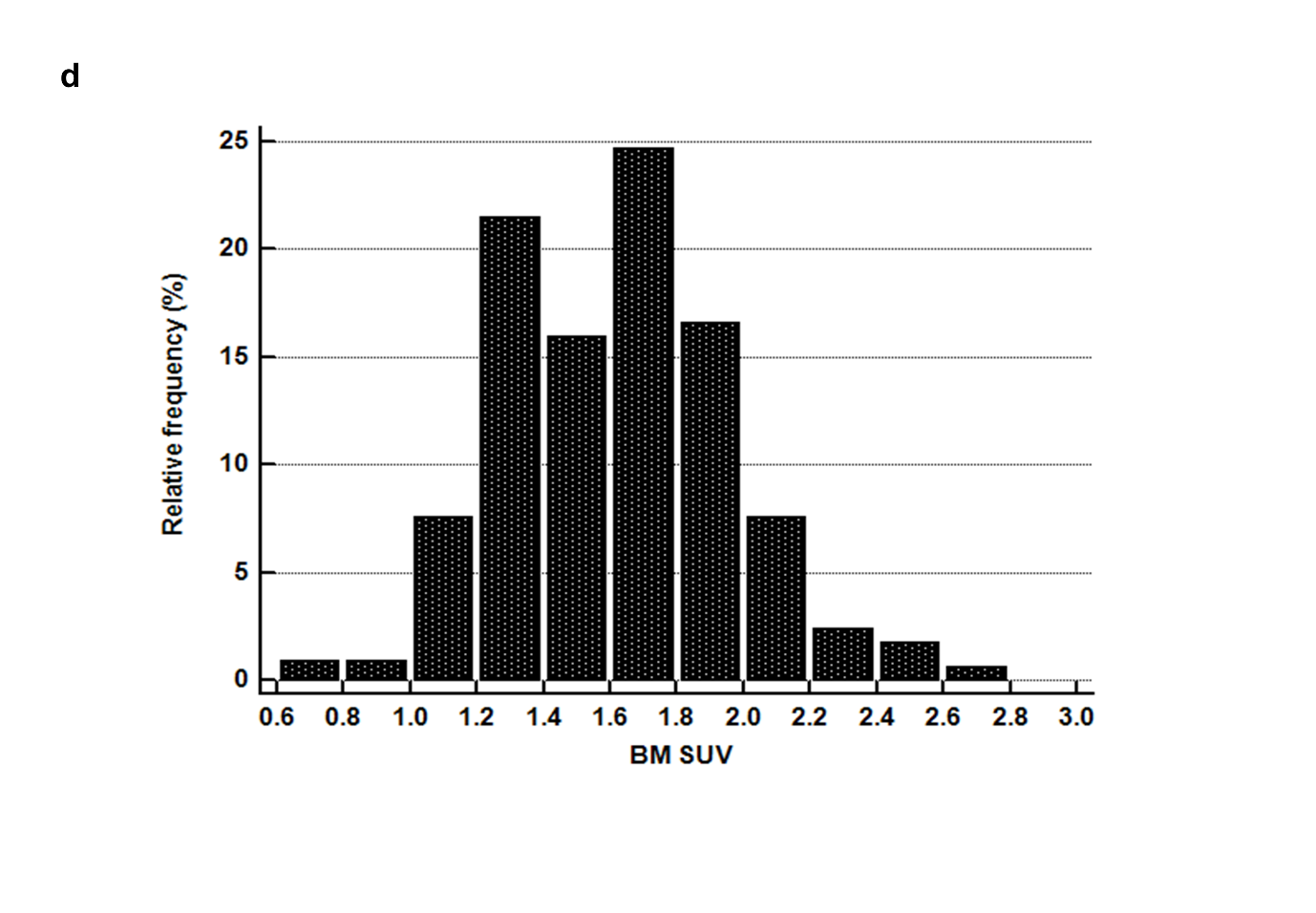


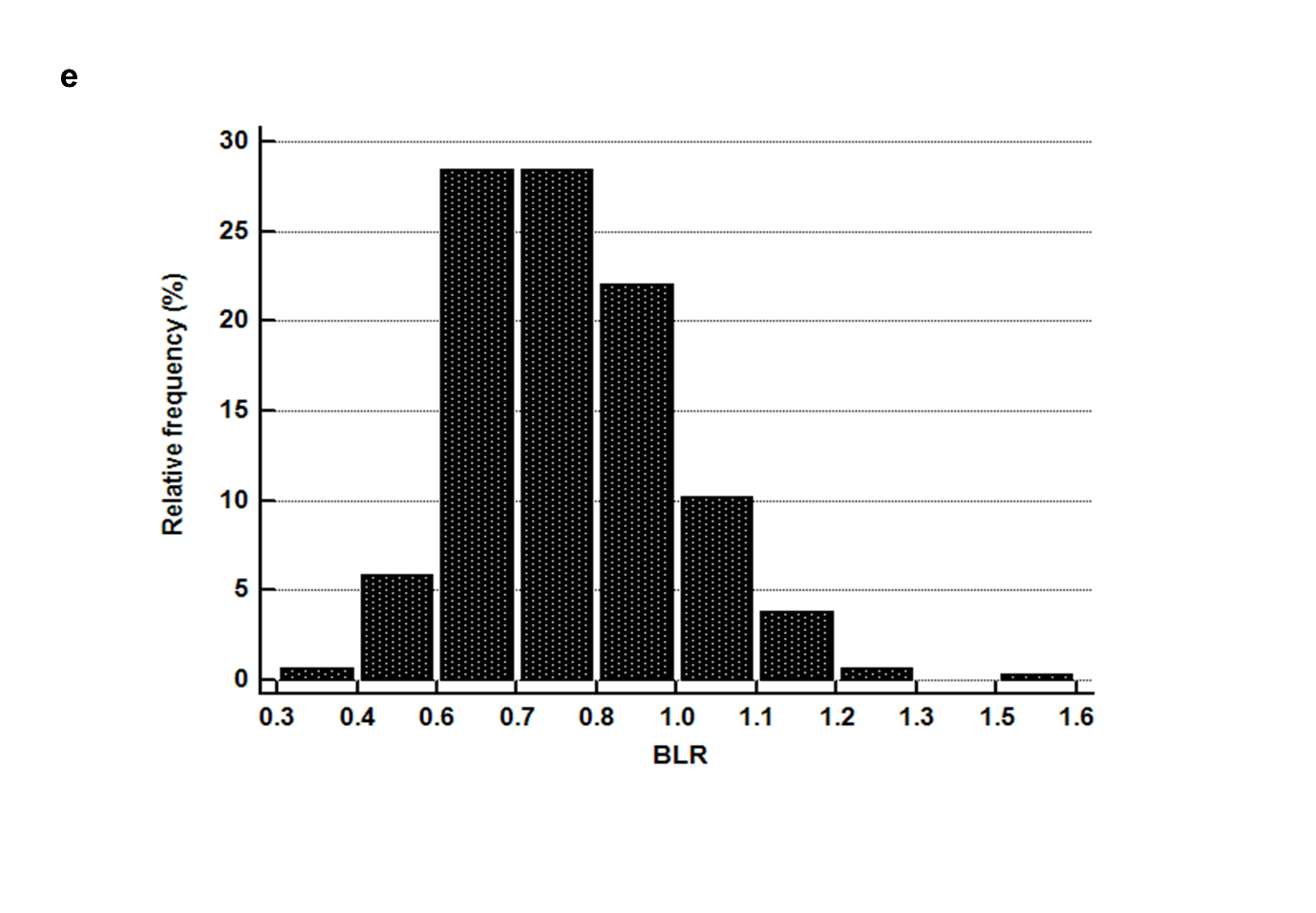

Supplement: Supplementary file 1 — Additional file 1: Figure 1. Histograms of maximum SUV (a), MTV (b), TLG (c), BM SUV (d), and BLR (e). [file 13550_2020_660_MOESM1_ESM.docx]
